# The diagnostic importance of invasive micropapillary carcinoma in the canine mammary gland: clinicopathological, immunohistochemical and survival analysis

**DOI:** 10.1186/1753-6561-7-S2-P17

**Published:** 2013-04-04

**Authors:** Conrado O Gamba, Éverton J Dias, Lorena GR Ribeiro, Liliane C Campos, Alessandra Estrela-Lima, Enio Ferreira, Geovanni D Cassali

**Affiliations:** 1 Department of General Pathology, Institute of Biological Sciences, Federal University of Minas Gerais, Belo Horizonte, Minas Gerais, Brazil; 2 Department of Pathology and Clinics, School of Veterinary Medicine, Federal University of Bahia, Salvador, Bahia, Brazil

## Background

Invasive micropapillary carcinoma (IMPC) of the mammary gland, despite its rare occurrence in humans and dogs, is an important neoplasm due to its aggressive behaviour. Our aim was to evaluate clinicopathological and immunophenotypical characteristics of IMPC and to determine overall survival of dogs with this tumour.

## Materials and methods

Twenty-two IMPC cases were selected for survival and clinicopathological analysis. Immunohistochemistry was performed for Epidermal Growth Factor Receptor (HER)-2, Epidermal Growth factor Receptor (EGFR), Oestrogen Receptor (ER), Progesterone Receptor (PR), CD-31, Cytokeratin (CK) AE1AE3, p63 and Epithelial Membrane Antigen (EMA).

## Results

Of the 22 studied cases, the majority had > 3 cm neoplasms (15/19, 78.95%) and lymph node metastases (16/16, 100%), but only two cases (2/9, 22.2%) had distant metastases. The IMPCs were classified as either pure (15/22, 68.18%) or mixed (7/22, 31.82%) types. A predominance of moderate histological grade (16 grade II) tumours was observed and the average overall survival was 120 days. Positive immunohistochemical staining for EMA and negative staining for CD-31, p63 and CK AE1AE3 in cystic formations confirmed the micropapillary nature of these neoplasms. The majority of cases were positive for RE (19/20, 95%) and RP (19/20, 95%), but lacked HER-2 (16/22, 72.72%) and EGFR (15/22, 68.18%) over-expression.

## Conclusions

These findings demonstrate that, similar to the situation pertaining with human IMPCs, canine IMPCs behave aggressively with high rates of metastasis to regional lymph nodes and short overall survival times.

## Financial support

FAPEMIG, CAPES and CNPq.

